# Examining the Integration of Biofeedback Treatment in Inpatient Mental Health Care, Specialized in Psychosomatic Medicine and Psychotherapy: A Mixed Methods Study

**DOI:** 10.1007/s10484-025-09749-3

**Published:** 2025-11-21

**Authors:** Gregor Gehrig, Tania Lalgi, Alexander Bäuerle, Martin Teufel, Kira Schmidt

**Affiliations:** 1 Clinic for Psychosomatic Medicine and Psychotherapy, LVR-University Hospital Essen, University of Duisburg-Essen, Essen, Germany; 2 Center for Translational Neuro- and Behavioral Sciences (C-TNBS), University of Duisburg-Essen, 45147 Essen, Germany

**Keywords:** Neurofeedback, Inpatient, Mental health, Mixed methods, Service structure

## Abstract

Although biofeedback (BFB) treatment has been well researched for different outpatient settings, relatively little information exists on the implementation process or the difficulties of applying this form of therapy in an inpatient setting. This study, therefore, focuses on the implementation of BFB in the inpatient setting and the potential barriers to its implementation and use. A mixed methods approach was chosen. Medical professionals in hospitals for inpatient mental health care, specializing in psychosomatic medicine and psychotherapy in Germany, were surveyed on the use of BFB via an online questionnaire. Hospitals that use BFB were then invited to take part in a semistructured interview. Participants were asked about the BFB treatment process and difficulties faced during implementation and application. Twenty-four hospitals participated in the quantitative part, and seven hospitals participated in the qualitative part of the study. The analysis of the data revealed that BFB implementation varied from hospital to hospital. The greatest differences were found primarily in the parameters surveyed, session frequency, and duration. Common features included the use of an introductory session, an evaluation, and a discussion of the benefits for patients. The obstacles included staff shortages, technical problems, and the high organizational effort required for BFB use. The data obtained confirm the benefits of BFB treatment in the inpatient setting and reveal which criteria are necessary for successful implementation. Sufficient resources (staff, time, equipment) must be provided for successful BFB implementation. Consistent integration into patients’ timetables could minimize organizational effort, and therapist training could improve handling and implementation.

**Supplementary Information:**

The online version contains supplementary material available at https://doi.org/10.1007/s10484-025-09749-3.

## Introduction

This study was conducted in German hospitals specializing in psychosomatic medicine and psychotherapy. Psychosomatic medicine is a distinct medical specialty in Germany that focuses on diagnosing, treating, preventing, and rehabilitating conditions in which psychosocial and psychophysiological factors substantially contribute to the onset and persistence of symptoms (Bundesärztekammer 2023). Care is delivered within a multimodal, multiprofessional framework that addresses physical and psychological symptoms equally. Typical indications include depressive disorders, somatoform disorders, posttraumatic stress disorder, and eating disorders. (Deutsche Gesellschaft für Psychosomatische Medizin und Ärztliche Psychotherapie (DGPM) e.V., n.d.).

Biofeedback (BFB) increases a patient’s awareness of emotions, thoughts, and physical sensations by translating unconscious physiological processes into visual or auditory stimuli (Haus et al., 2016). This provides real-time feedback and supports the development of self-regulatory skills. Common modalities include EMG (electromyogram), ECG (electrocardiogram), HRV (heart rate variability), EDA (electrodermal activity), and EEG (electroencephalography), the latter known as neurofeedback (NFB). BFB utilizes learning mechanisms, specifically operant conditioning, by reinforcing desired physiological states. For example, maintaining HRV within a target range associated with relaxation is supported by the presence or absence of feedback signals that prompt patients to consciously restore balance when deviations occur (Frank et al., 2010). By introducing novel sensory inputs, BFB stimulate both conscious and unconscious learning processes and enhance the ability of the brain to modulate previously inaccessible physiological functions (Haus et al., 2016).

Several studies indicate that BFB can alleviate symptoms of mental disorders and support therapeutic success. HRV-based BFB have been shown to reduce depressive symptoms in patients with various psychophysiological conditions (Pizzoli et al., 2021) and in those diagnosed with major depressive disorder (Fernández-Alvarez et al., 2022). In patients with complex posttraumatic stress disorder (PTSD), BFB can reduce symptoms such as anxiety, arousal, numbness, suicidal ideation, and intrusive thoughts (Choi et al., 2023). It has also been associated with an increased likelihood of long-term remission in PTSD patients (Askovic et al., 2023). A randomized controlled trial (RCT) pilot study demonstrated that neurofeedback significantly reduced eating disorder psychopathology, food cravings, and binge episodes in patients with binge eating disorders (Blume et al., 2022). Most research on BFB has been conducted in outpatient settings, and there are limited data on its use in inpatient mental health care settings. Although the German Hospital Association reports that 42 such hospitals offer BFB (Deutsche Krankenhaus Gesellschaft e.V., n.d.), this claim often contradicts information on hospitals’ own websites, leaving actual availability unclear. An inpatient study revealed that BFB was well received by both patients and therapists but also highlighted barriers to implementation, including technical issues, limited therapist experience, and time constraints (Schmidt et al., 2023). To develop recommendations for the effective integration of BFB into inpatient care, this study aims to explore current practices and challenges in different hospitals for psychosomatic medicine and psychotherapy.

## Methods

To determine whether and how BFB treatment is used and what potential challenges may arise during implementation, we chose a two-step approach involving a quantitative survey followed by qualitative interviews. This made it possible to reach as many hospitals as possible and gain detailed insight into how BFB is conducted in individual hospitals at the same time. This study adhered to the Mixed Methods Article Reporting Standards (MMARS) issued by the American Psychological Association (APA) (see Supplementary Material A1) (Levitt et al., 2018).

### Study Design

Hospitals were selected across Germany on the basis of the German Hospital Association registry (Deutsche Krankenhaus Gesellschaft e.V., n.d.). The inclusion criteria required the hospitals to specialize in psychosomatic medicine and psychotherapy. Additionally, only hospitals that offered inpatient care were included. Hospitals that focused on rehabilitation were excluded. A survey was developed for the quantitative component to collect data on BFB treatment practices. For the qualitative component, hospitals offering BFB were invited to participate in interviews for further insights. The participating hospitals received an invitation email, and interviews were scheduled with relevant medical staff and chief physicians.

### Data Collection

Data were collected from March to July 2024, with the interviews for the qualitative part conducted at the end of this period. The quantitative data were surveyed online via Unipark (Tivian, Köln) survey software. The characteristics of the hospitals were collected from the official website of the German Hospital Association (Deutsche Krankenhaus Gesellschaft e.V., n.d.). The online survey asked the respective hospitals which patient groups BFB was administered to as well as the general conditions for BFB treatment (premises, staff, type of BFB treatment). The questionnaire can be found in Supplementary Material A2. Difficulties in the implementation of BFB in clinical practice were also surveyed. Hospitals that do not use BFB were asked during the survey why BFB had not become part of their therapy program.

To collect valuable qualitative data regarding BFB use, semistructured interviews were conducted online via Zoom (Zoom Communications, San Jose, CA). The interview guide contained eight open-ended questions to which the participants could respond freely. These interviews were audio recorded with the record function of Zoom after participants were informed about the recording of the interview and gave their verbal consent. On average, each interview lasted 30 min. At least one representative from each hospital participated in the interview. These individuals are responsible for performing BFB treatments in their respective hospitals. In some cases, chief physicians or deputy senior physicians participated as additional interview partners. The respondents were from professional groups, including nurses, medical assistants, psychotherapists, and physicians. The qualitative part of our analysis addresses in greater detail the anecdotal accomplishments of BFB, as well as problems experienced with its implementation and realization. The first step was to determine how long BFB had been used at the hospital and the reasons for its implementation. During the survey, questions were asked about difficulties in the implementation process, as well as difficulties in implementation in everyday clinical practice. The procedure of a BFB session, the preparation of the patients for BFB sessions, and the evaluation after the sessions were also discussed. At the end of the interview, the acceptance of BFB treatment by the therapeutic team and among patients was recorded. Moreover, the subjective benefit of BFB for patients from the interviewees’ point of view was investigated.

### Participants

**Fig. 1:**
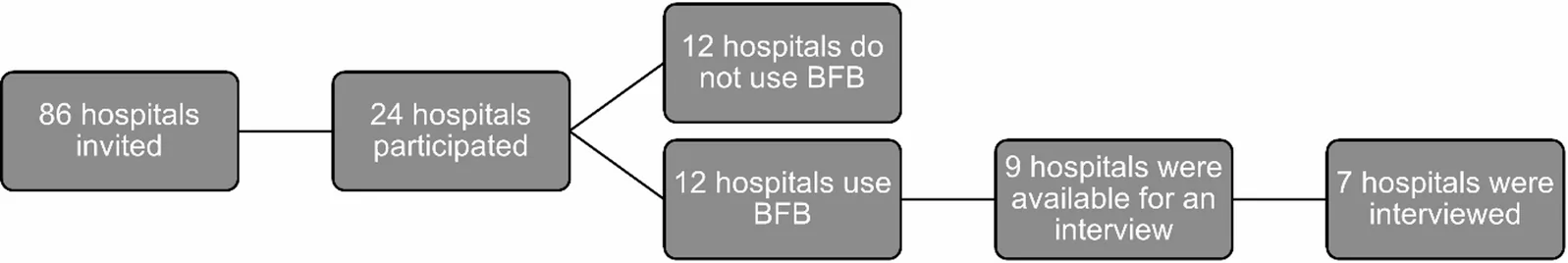
Flowchart depicting the selection process

The survey was sent to 86 hospitals in Germany (see Fig. 1). In total, 24 hospitals participated in the survey (response rate 27.9%). All of the participating hospitals had at least one inpatient department that specialized in psychosomatic medicine and psychotherapy. For anonymization purposes, the term "hospital" is generally used. All of the hospitals employed a multimodal, multiprofessional treatment approach. Inpatient care is typically delivered by interdisciplinary teams consisting of medical specialists in psychosomatic medicine and psychotherapy, psychologists, nurses, occupational therapists, physical therapists, social workers, and sometimes creative arts therapists and dietitians. Treatment concepts integrate evidence-based psychotherapeutic methods with somatic interventions such as physiotherapy, movement therapy, occupational therapy, art therapy, and dietary counseling. These interventions aimed to address the psychological and physical dimensions of illness. For more hospital characteristics, see Table 1. BFB treatment was offered in 12 of the responding hospitals. On average, the included hospitals treated 206.91 inpatient cases (SD = 129.116, range = 80–571) per year in the fields of psychosomatic medicine and psychotherapy (Deutsche Krankenhaus Gesellschaft e.V., n.d.).

**Table 1:** Hospital characteristics

	n	Percentage (%)
*Type of hospital*		
University hospital	9	37.5
Regular hospital	14	58.3
Missing	1	4.2
*Total bed capacity (including all departments)*		
> 1000	10	41.7
400–1000	10	41.7
< 400	4	16.6
*Hospital operator*		
State	10	41.7
Municipal	8	33.3
Church or social foundation	5	20.8
Private	1	4.2
*Federate State*		
Brandenburg	1	4.2
Sachsen	1	4.2
Sachsen-Anhalt	1	4.2
Mecklenburg-Vorpommern	1	4.2
Nordrhein-Westfalen	2	8.3
Baden-Württemberg	8	33.3
Berlin	2	8.3
Bayern	4	16.7
Hamburg	1	4.2
Schleswig–Holstein	1	4.2
Niedersachsen	1	4.2
Missing	1	4.2
*BFB treatment*		
Yes	12	50.0
No	12	50.0

*N* = 24

### Data Analysis

The anonymized questionnaires were analyzed via SPSS version 26 (IBM, Armonk, NY). Frequencies were calculated for all the quantitative questionnaire data. For the qualitative data, the recorded interviews were transcribed via f4x speech recognition software (Audiotranskription, 2024). The transcribed data were analyzed via MAXQDA (2024) via the thematic analysis method according to the concept developed by Braun and Clarke (Braun & Clarke, 2006). Two independent researchers (K.S. and G.G.) divided the content of the transcripts into codes that were consistent for them and then compared them with each other to develop a uniform code structure. Next, the codes were again divided into themes by a third independent researcher (T.L.) and the first author (G.G.). These were discussed and standardized in a plenary session of the research team. The codes were once again reevaluated to ensure their relevance to the defined themes and subthemes. The codes were then assigned to the appropriate themes and subthemes to create the final codebook (the codebook was translated verbatim for publication reasons and is shown in Supplementary Material A4). The contents of the transcribed interviews can only be quantified to a limited extent in the opinion of the research team, owing to the small number of seven interviews. Additionally, the sometimes strong deviations in the approach to BFB treatment at hospitals led to the choice of the rather open approach described (Creswell & Poth, 2025). This allowed the various experiences of individual hospitals, as well as their similarities and differences, to be identified, resulting in potential recommendations for dealing with BFB in the inpatient setting.

### Quality Control

This study relies on the Mixed Methods Article Reporting Standards (MMARS) issued by the American Psychological Association (Levitt et al., 2018). Independent coding by two independent researchers (K.S. and G.G.) and cross-coding by a third independent researcher (T.L.) increased the credibility of the data. The research group consisted of PhD candidates and graduate students with varying levels of experience in quantitative and qualitative research. The interviews were conducted by a neutral researcher with practical experience in BFB treatment. The interviewed hospitals participated voluntarily in the study and had no relationship with the research team. To increase credibility, the methodology details the conditions under which the qualitative study section was conducted, including information on the interviewees, the study design, and the procedures (Hussy et al., 2013). To ensure the validity of the study, Creswell and Miller’s validity guidelines were used (Creswell & Miller, 2000).

## Results

### Quantitative Online Questionnaires

#### Results of Hospitals that use BFB

Table 2 presents comprehensive data from hospitals that conduct BFB treatment. The information provided by these hospitals relates to the areas of application and implementation of BFB treatment, structural conditions (e.g., facilities, professional groups using the treatment, equipment used), and obstacles encountered during implementation.

**Table 2:** Characteristics of BFB treatment at surveyed hospitals

	n	Percentage (%)
*Type of setting*		
Inpatient	9	75.0
Inpatient and Outpatient	3	25.0
*Treated mental health disorders with BFB*		
Posttraumatic stress disorders	4	33.3
Depressive disorders	7	58.3
Somatoform disorders	12	100
Anxiety disorders	9	75.0
Panic disorders	8	66.7
Eating disorders	5	41.7
Psychotic disorders	1	8.3
Obsessive–compulsive disorders	3	25.0
Post-Covid syndrome	1	8.3
Tinnitus	1	8.3
*Exclusion criteria*		
Everyone could take part	3	25.0
Lack of compliance	2	16.7
Lack of German language	2	16.7
Length of inpatient stay	5	41.7
Comorbid disorders	0	0
Limited cognitive abilities	3	25.0
Limited visual abilities	2	16.7
Hypacusis/deafness	1	8.3
Other	4	33.3
*BFB parameter*		
Electroencephalography	2	16.7
Electromyography	9	75.0
Electrocardiography	9	75.0
Electrodermal activity	10	83.3
Respiratory frequency	8	66.7
Other	1	8.3
*Device*		
Nexus	4	33.3
Mindfield	2	16.7
Other	7	58.3
*Profession*		
Nurses	5	41.7
Occupational therapists	1	8.3
Physicians	5	41.7
Psychotherapists	5	41.7
Other	7	58.7
*Advanced training*		
Yes	8	66,7
No	4	33,3
*Premises*		
BFB room	9	75.0
Patient room	0	0
Group room	1	8.3
Occupational therapist room	0	0
Single room	3	25.0
Other	1	8.3
*Average number of sessions per patient per stay*		
1–5	7	58.3
6–10	3	25.0
11–15	2	16.7
*Length of sessions*		
< 20 min	2	16.7
20–30 min	4	33.3
31–45 min	2	16.7
> 45 min	4	33.3
*Number of patients per week*		
0–5	8	66.7
6–10	1	8.3
11–15	2	16.7
> 15	1	8.3
*BFB protocol*		
Yes	4	33.3
No	8	66.7
*Evaluation*		
Yes, after every session	3	25.0
Yes, before and after every session	6	50.0
Yes, before and after BFB treatment	1	8.3
Yes, intermittently	2	16.7
*Difficulties*		
Staff shortage	9	75.0
Lack of time	10	83.3
Accounting	3	25.0
Adherence	1	8.3
Technical problems	3	25.0
Premises	2	16.7
*Interview*		
Yes	9	75.0
No	3	25.0

*N* = 12

Table 3 categorizes the characteristics of Table 2 according to the various BFB modalities used by the surveyed hospitals (EEG, EMG, ECG, EDA, and respiratory frequency (RF)). This provides insight into how the different modalities may influence the implementation and execution of BFB treatment.

**Table 3:** Characteristics of BFB treatment at the surveyed hospitals, based on the BFB modality used

	EEG (*N* = *2*)		EMG (*N* = *9*)		ECG (*N* = *9*)		EDA (*N* = *10*)		RF (*N* = *8*)	
	n	%	n	%	n	%	n	%	n	%
*Treated mental health disorders with BFB*										
Posttraumatic stress disorders	1	50	2	22.2	3	33.3	3	30	2	25
Depressive disorders	1	50	5	44.4	6	66.7	6	60	5	62.5
Somatoform disorders	2	100	9	100	9	100	10	100	8	100
Anxiety disorders	2	100	6	66.7	7	77.8	7	70	6	75
Panic disorders	2	100	5	55.6	6	66.7	6	60	5	62.5
Eating disorders	0	0	3	33.3	4	44.4	4	40	3	37.5
Psychotic disorders	0	0	0	0	1	11.1	0	0	1	12.5
Obsessive–compulsive disorders	1	50	1	11.1	1	11.1	2	20	1	12.5
*Exclusion criteria*										
Everyone could take part	0	0	3	33.3	3	33.3	3	30	2	25
Lack of compliance	1	50	1	11.1	1	11.1	1	10	1	12.5
Lack of German language	1	50	1	11.1	1	11.1	1	10	1	12.5
Length of inpatient stay	0	0	3	33.3	3	33.3	4	40	4	50
Limited cognitive abilities	0	0	3	33.3	3	33.3	3	30	3	37.5
Limited visual abilities	1	50	1	11.1	1	11.1	1	10	1	12.5
Hypacusis/deafness	0	0	1	11.1	1	11.1	1	10	1	12.5
*Profession*										
Nurses	0	0	5	55.6	5	55.6	5	50	4	50
Occupational therapists	1	50	1	11.1	1	11.1	1	10	0	0
Physicians	1	50	5	55.6	4	44.4	5	50	3	37.5
Psychotherapists	1	50	4	44.4	3	33.3	5	50	3	37.5
*Advanced training*										
Yes	2	100	6	66.7	7	77.8	6	60	6	75
No	0	0	3	33.3	2	22.2	4	40	2	25
*Premises*										
BFB room	2	100	6	66.7	8	88.9	7	70	6	75
Group room	0	0	1	11.1	0	0	1	10	0	0
Single room	0	0	2	22.2	1	11.1	2	20	2	25
*Average number of sessions per patient per stay*										
1–5	1	50	6	66.7	6	66.7	7	70	4	50
6–10	0	0	3	33.3	2	22.2	3	30	3	37.5
11–15	1	50	0	0	1	11.1	0	0	1	12.5
*Length of sessions*										
< 20 min	0	0	1	11.1	2	22.2	1	10	1	12.5
20–30 min	0	0	4	44.4	3	33.3	4	40	4	50
31–45 min	0	0	2	22.2	2	22.2	2	20	2	25
> 45 min	2	100	2	22.2	2	22.2	3	30	1	12.5
*Number of patients per week*										
0–5	2	100	6	66.7	5	55.6	7	70	4	50
6–10	0	0	1	11.1	1	11.1	1	10	1	12.5
11–15	0	0	2	22.2	2	22.2	2	20	2	25
> 15	0	0	0	0	1	11.1	0	0	1	12.5
*BFB protocol*										
Yes	1	50	2	22.2	2	22.2	2	20	2	25
No	1	50	7	77.8	7	77.8	8	80	6	75
*Evaluation*										
Yes, after every session	1	50	2	22.2	3	33.3	2	20	2	25
Yes, before and after every session	1	50	4	44.4	3	33.3	5	50	4	50
Yes, before and after BFB treatment	0	0	1	11.1	1	11.1	1	10	1	12.5
Yes, intermittently	0	0	2	22.2	2	22.2	2	20	1	12.5
*Difficulties*										
Staff shortage	2	100	7	77.8	6	66.7	8	80	5	62.5
Lack of time	2	100	8	88.9	7	77.8	9	90	7	87.5
Accounting	0	0	3	33.3	3	33.3	3	30	3	37.5
Adherence	0	0	1	11.1	1	11.1	1	10	1	12.5
Technical problems	1	50	2	22.2	2	22.2	3	30	0	0
Premises	0	0	1	11.1	0	0	2	20	1	12.5

*N* = *12*

Since most hospitals use multiple modalities, the following list shows how many hospitals use each modality:

EMG, ECG, EDA, and AF are used in 50% (n = 6) of hospitals. EEG, EMG, ECG, and EDA are used by 8.33% (n = 1) of hospitals, as are EMG, ECG, and EDA (8.33%, n = 1) and EMG, EDA, and AF (8.33%, n = 1). ECGs and AFs were used by 8.33% (n = 1) of the hospitals. EEG alone is used in 8.33% (n = 1) of hospitals, and the same applies to the use of EDA (8.33%, n = 1) as the primary modality.

All hospitals (100%, *n* = 12) utilized BFB for patients with somatoform disorders. Of these, 83.3% (*n* = 10) use EDA BFB as a feedback mechanism. The most commonly reported barrier to implementing BFB treatments in daily clinical practice is lack of time, reported by 83.3% (*n* = 10) of the hospitals.

Other exclusion criteria for taking part in BFB treatment were PTSD (8.3%, *n* = 1), limited capacity (8.3%, *n* = 1), other therapeutic reasons, e.g., excessive introspection (8,3%, *n* = 1) and individual reasons (8.3%, *n* = 1).

Another BFB parameter that is trained is respiratory sinus arrhythmia (8.3%, *n* = 1). Other devices used for BFB treatment included the NeuroAmp II (8.3%, *n* = 1), the BioSign HRV-Scanner Professional (8.3%, *n* = 1), Insight (16.7%, n = 2), Xpert (8.3%, *n* = 1), Neuromaster (16.7%, n = 2), and Qiu (8.3%, *n* = 1) devices.

Other professionals who were conducting BFB treatment in the surveyed hospitals included medical assistants (16.7%, *n* = 2), psychologists (8.3%, *n* = 1), physiotherapists (16.7%, *n* = 2), and body therapists (8.3%, *n* = 1). Other rooms used for BFB treatment were rooms for medical rounds (8.3%, *n* = 1). The surveyed hospitals offered BFB at different points in time. In some cases, BFB has been part of the therapy program for 20 years, whereas in others, it has been newly implemented.

#### Results of Hospitals that do not use BFB

Table 4 presents data from the surveyed hospitals that do not offer BFB treatment. The primary question was about the reasons for not offering BFB. Seventy-five percent (*n* = 9) of the hospitals reported a lack of knowledge about treatment as a key factor. In addition, hospitals were asked what they thought was necessary to successfully implement the treatment and whether they could envision adding BFB to their practices in the near future.

**Table 4:** Reasons for not conducting BFB

	n	Percentage (%)
*Reasons against implementation*		
Lack of knowledge	9	75.0
Finances/Accounting	7	58.3
Staff shortage	8	66.7
Lack of premises	7	53.8
Lack of suitable patient clientele	0	0
Lack of time	6	50.0
Other	1	8.3
*Requirements for implementation*		
Increase in staff	5	41.6
Suitable room	3	25.0
Financial resources	2	16.7
Advanced training	5	41.6
*Implementation realizable*		
Yes	4	33.3
No	8	66.7

*N* = 12

### Qualitative Interviews

In the quantitative study, all twelve hospitals that used BFB were invited to participate in the qualitative section. Nine hospitals (75%) agreed, and seven interviews (58,3%) were successfully conducted. Table 5 presents relevant quantified data from these interviews, which are not included in the codebook but are considered important by the research team.

**Table 5:** Additional information regarding BFB treatment at the interviewed hospitals

	n	Percentage (%)
*Number of years since BFB has been used*		
≤ 1 year	1	14.0
2–3 years	1	14.0
4–5 years	1	14.0
6–8 years	2	29.0
> 8 years	2	29.0
*Reasons for implementing BFB*		
Initiative of employee or head physician	4	57.0
Visualization of the connections between physical symptoms and psyche	3	43.0
Additional offer for patients with chronic pain	1	14.0
Additional offer to support patients in improving their self-perception	1	14.0
Positive experience reports from other hospitals	1	14.0
*Acceptance of BFB treatment by the team*		
Positive	5	71.0
Not truly present in the team	2	29.0
*Acceptance of BFB treatment by patients*		
Predominantly positive	7	100.0
Partial initial skepticism	3	43.0
Patients rarely reject the therapy	1	14.0

*N* = 7

### BFB Treatment Process

All the participating hospitals were asked about their implementation of BFB treatment. Appendix A3 summarizes their responses, which are categorized into four stages: (1) introduction to BFB treatment, (2) performing the treatment, (3) evaluation with patients, and (4) transfer of BFB to patients’ day-to-day lives. Reported feedback mechanisms and therapist qualifications were also documented. While implementation varied across hospitals, recurring patterns were identified and discussed below.

1. Introduction to BFB treatment
  Five hospitals (71.4%) reported conducting an initial consultation to explain the concept and procedure of BFB therapy prior to the first session. Baseline measurements at rest were conducted by three hospitals (42.9%), and an equal number of hospitals performed stress tests involving cognitive tasks to obtain comparative data. These assessments were performed either separately or in conjunction with the introductory session. In addition, three hospitals (42.9%) provided patients with informational brochures outlining key aspects of BFB treatment.
2. Performing the treatment
  The application of BFB treatment varies considerably across hospitals and is influenced by treatment goals and selected feedback mechanisms. Because four hospitals (57.1%) reported adapting the application to individual patient needs, Appendix A3 includes only explicitly described procedures. All hospitals (100%) used visual feedback; four (57.1%) provided real-time graphical displays (e.g., ECG, EMG) with explanatory guidance. Three hospitals (42.9%) used visual cues that changed on the basis of patient performance. Breathing exercises were used in four hospitals (57.1%), whereas other methods included discrimination training, relaxation techniques, muscle relaxation, neurofeedback (NFB), and self-awareness training. Session duration varied widely, with most hospitals (57.1%) reporting treatment times of up to 50 min.
3. Evaluation with the patients
  All seven hospitals (100%) reported having an established evaluation process. For five (71.4%) hospitals, each session was evaluated from both the patient and therapist perspectives. Three hospitals (42.9%) regularly reviewed treatment progress, allowing patients to reflect on improvements and possible setbacks. Two hospitals (28.6%) emphasized joint reflection on states of relaxation and tension during the sessions.
4. Transfer of BFB to patients’ day-to-day lives
  This section examined how patients were supported in applying BFB techniques in their day-to-day lives. The emphasis was placed on the manner in which inpatient instructional and practical opportunities were conceptualized to empower patients to extend the benefits of BFB beyond the confines of the treatment environment and seamlessly integrate them into their daily lives in a sustainable way. Four hospitals (57.1%) assigned exercises between sessions that were regularly reviewed by therapists. Two hospitals (28.6%) intentionally omitted device-based feedback at certain stages to encourage the independent use of learned strategies. In three hospitals (42.9%), no transfer process was performed or explicitly described.

### Themes Discovered

**Fig. 2:**
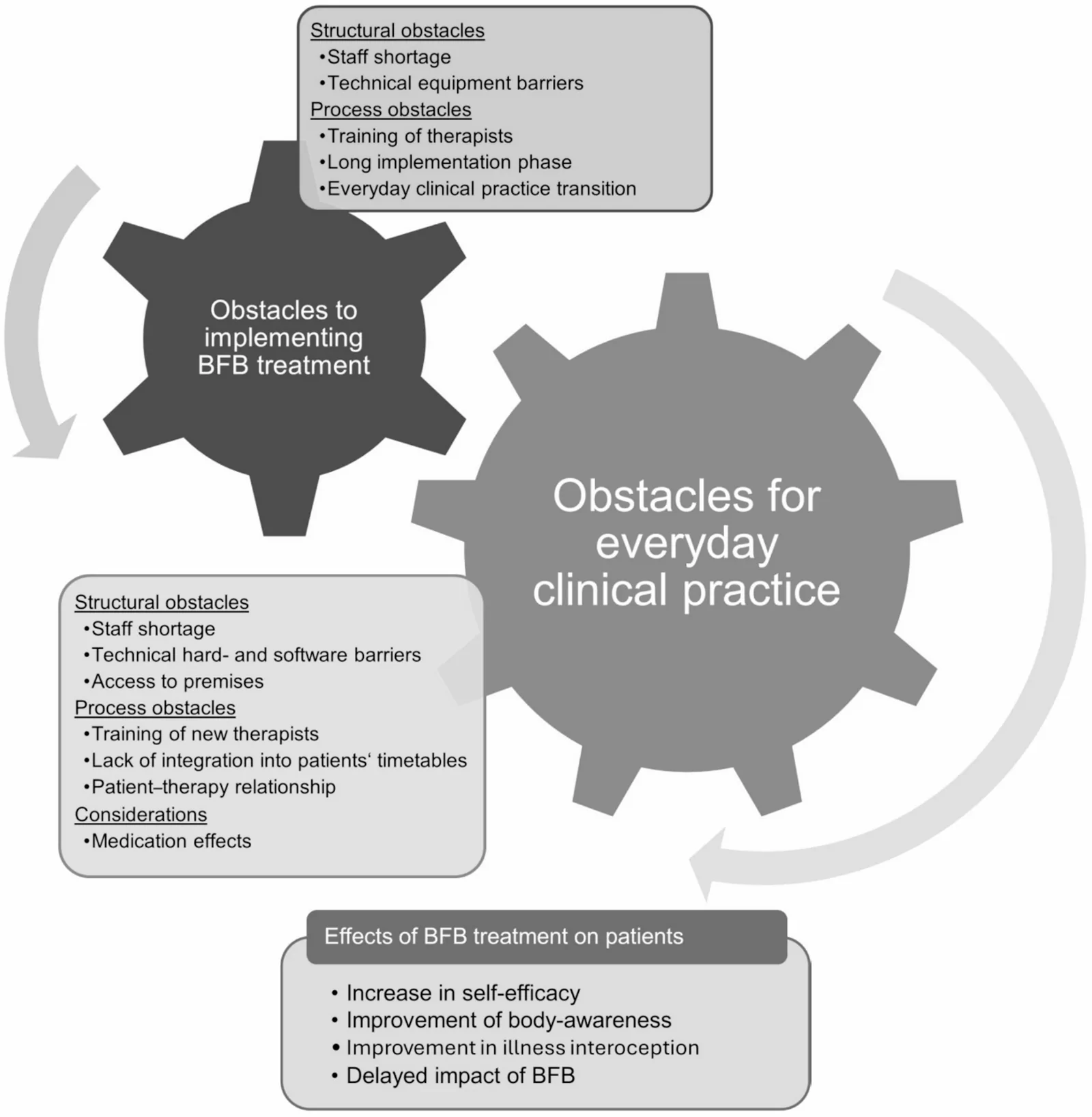
A graphic display of the overarching themes and subthemes during the iterative thematic analysis of BFB implementation, which is based on the codebook analysis

Three major categories were identified: (1) obstacles to implementing BFB treatment (with five subthemes), (2) obstacles for everyday clinical practice (with seven subthemes), and (3) effects of BFB treatment on patients (with four subthemes). These categories were structured on the basis of the Donabedian quality model (Donabedian, 1988), with the first two categories further subdivided into structural and process barriers and an additional theme, Considerations, to address subthemes that do not fit into the other categories. The third category focuses on outcome quality. A visual representation of these themes and subthemes is provided in Fig. 2, with corresponding quotes in Appendix A4.

#### Category 1—Obstacles to Implementing BFB Treatment

This category addresses the various problems that can arise when introducing BFB treatment in an inpatient setting. Structural obstacles and obstacles to various processes are considered here.

*Theme 1. Structural obstacles*

*Subtheme 1.1 – Staff shortages*

Staff shortages were identified as a major barrier by four hospitals (57.1%) (see Appendix A4, [Q1]). One hospital reported that plans for nursing staff to assume responsibility for BFB treatment were hindered by staff shortages, illness, and rotation [Q2]. Another hospital noted that implementation had failed due to unfilled positions and a lack of ownership among nursing staff [Q3].

*Subtheme 1.2—Technical equipment barriers*

Four hospitals (57.1%) reported difficulties with the specialized equipment required for BFB treatment. Challenges included identifying appropriate devices, electrodes, and software [Q4] and increasing the complexity of the procedure [Q5]. One hospital also noted the high cost of acquiring hardware and software for specific BFB applications [Q6].

*Theme 2. Process obstacles*

*Subtheme 2.1—Training of therapists*

Two hospitals (28.6%) identified therapist training as a challenge [Q7]. One hospital noted that the complexity of the training made implementation difficult, particularly as nurses were expected to provide BFB treatment [Q8].

*Subtheme 2.2—Long implementation phase*

Two hospitals (28.6%) commented on the implementation process. One reported slow progress [Q9], while another described the process as increasingly complex, noting that it took two years to finalize decisions about treatment responsibilities, equipment setup, and room preparation [Q10].

*Subtheme 2.3—Everyday clinical practice transition*

Two hospitals (28.6%) reported difficulties in integrating BFB treatment into daily practice. One hospital mentioned that although BFB was part of the treatment concept, it could not be integrated into routine work [Q11]. Another cited organizational problems that prevented the implementation of its original plan [Q12].

#### Category 2—Obstacles for Everyday Clinical Practice

This category examines the structural and procedural barriers to providing BFB treatment in an inpatient setting, as well as other relevant considerations.

*Theme 1. Structural obstacles*

*Subtheme 1.1—Staff shortage*

Five hospitals (71.4%) reported that staff shortages limited or prevented BFB treatment. Therapists were often responsible for multiple tasks, which deprioritized BFB treatment [Q13] or limited the number of sessions that could be provided [Q14]. In some cases, treatment was entirely dependent on one therapist, and absences due to illness or vacation interrupted BFB sessions [Q15, Q16]. Recurrent staff shortages were also cited as disrupting the treatment process [Q17].

*Subtheme 1.2—Technical hard and software barriers*

Three hospitals (42.9%) reported various technical issues. One hospital noted that the program often did not run reliably [Q18]. Two hospitals experienced problems with outdated technology, including insufficient storage capacity, which required repeated erasure of patient data [Q19]. Another hospital experienced problems with battery and electrode connections due to the age of the equipment [Q20].

*Subtheme 1.3—Access to premises*

Two hospitals (28.6%) reported difficulties in finding suitable rooms for BFB treatment. In one hospital, patients had to perform BFB in semipublic areas after receiving therapist instructions, which caused patient discomfort [Q21]. In another hospital, the designated BFB room was often occupied by other therapies, requiring the use of a mobile device. This added the challenge of confirming room availability before each session [Q22, Q23].

*Theme 2. Process obstacles*

*Subtheme 2.1—Training of new therapists*

Training new therapists was problematic, as reported by two (28.57%) hospitals. This is due mainly to constant changes and rotations in the therapeutic team [Q24, Q25].

*Subtheme 2.2—Lack of integration into patients’ timetables*

Five hospitals (71.4%) reported organizational challenges because BFB were not an integral part of patients’ treatment plans. One hospital noted that although gaps in the treatment plan were used for BFB, scheduling conflicts often resulted in postponements [Q26, Q27]. Others reported difficulties integrating BFB into existing treatment schedules [Q28, Q29], resulting in BFB being treated as secondary [Q30] or used less frequently than intended [Q31].

*Subtheme 2.3—Patients–therapy relationship*

Six hospitals (85.7%) reported instances of patient resistance to BFB treatment. Some patients were skeptical, often because of past experiences. One hospital treated individuals with a Stasi history who initially expressed safety concerns and required several sessions to build trust [Q32]. Others noted that anxious patients were unsettled by sensors and electrodes [Q33, Q34]. Difficulties with patient cooperation were also mentioned, including distractibility—requiring screen adjustments to maintain focus [Q35]—and irregular participation, sometimes attributed to general resistance to therapy [Q36].

*Theme 3. Consideration*

*Subtheme 3.1—Medication effects*

Two hospitals (28.6%) reported that certain medications affected BFB results and required adjusted data interpretation [Q37]. Beta-blockers were specifically noted to affect HRV measurements [Q38], with changes in treatment observed following dose adjustments [Q39].

#### Category 3—Effects of BFB Treatment on Patients

This category addresses the effects of BFB treatment on patients from the perspective of the hospitals surveyed.

*Theme 1. Increased self-efficacy*

Six hospitals (85.7%) reported improvements in patient self-efficacy. Three (42.9%) emphasized improved relaxation skills, including patients who reported relaxing after prolonged difficulty [Q40]. It was also mentioned that patients experienced the effects of BFB treatment outside of the sessions. These effects included a reduced need for staff support [Q41] and increased relaxation awareness [Q42]. One hospital described a patient using BFB techniques to manage a panic attack [Q43], whereas another reported that long-term use of BFB exercises contributed to sustained well-being [Q44].

*Theme 2. Improvement of body awareness*

Five hospitals (71.4%) reported that BFB treatment improved patients’ body awareness and helped reduce tension [Q45]. Patients with chronic neck pain gain insight into the effects of tension [Q46], and patients with anxiety develop a better ability to recognize and manage rising tension levels [Q47].

*Theme 3. Improvement in illness interoception*

Five hospitals (71.4%) reported that BFB helped patients better understand the causes of their symptoms. For example, patients with chronic pain often become aware of previously unnoticed muscle tension, allowing discussion of its possible role in their condition [Q48, Q49]. In one case, BFB helped a patient realize that his distress was due to rumination rather than physical tension [Q50]. The visualization of symptoms through data was found to be highly beneficial for some patients [Q51]. Particularly for somatoform disorders, BFB facilitates "aha" moments, helping patients connect physiological data with internal experiences [Q52, Q53].

*Theme 4. Delayed impact*

Four hospitals (57.1%) noted that the effects of BFB varied from patient to patient. One hospital emphasized that, as with any therapy, the results varied in the degree of benefit [Q54]. Another reported that some patients experienced delayed effects, with improvements taking several weeks [Q55]. In addition, some patients require extended therapeutic support, particularly in identifying emotional states [Q56].

## Discussion

This study investigated how BFB treatment is implemented in hospitals for psychosomatic medicine and psychotherapy in Germany and what challenges arise during its introduction and everyday clinical use. The data revealed a high degree of heterogeneity in BFB practices across hospitals, particularly with respect to treatment procedures and the physiological parameters measured (e.g., EMG, ECG, and EEG). Hospitals differ in their introductory protocols: some conduct stress tests, others conduct baseline measurements, some conduct both, and others conduct only an initial interview. The frequency and duration of sessions vary widely, from once to several times per week and from a few minutes to more than an hour.

The use of BFB also varies in function: it is used as a self-regulation skill, as a tool for teaching disorder models, or as an extension of behavioral therapy. Approaches to integrating BFB into daily life also vary, with some hospitals teaching exercises that are consistent with BFB techniques, such as breathing or relaxation training. Treatment is provided by a variety of professionals, including nurses, psychotherapists, and physicians.

Despite these differences, there are striking similarities. All hospitals conduct some form of introductory session to reduce initial patient skepticism, a commonly reported concern. Stress and baseline tests help assess patients’ initial status and allow for more accurate tracking of progress. Evaluation is standard across hospitals, often after each session, and typically includes therapist‒patient discussions aimed at improving patients’ ability to recognize and reflect on their physical and emotional states. Visual feedback is universally used, although the type and format vary from images that sharpen as targets are reached to dynamic graphs of physiological data.

The reported benefits of BFB include increased self-efficacy in coping with stress, improved self-awareness, and greater insight into illness. The objective visualization of psychological symptoms (e.g., pain caused by tension or heightened arousal) appears to be particularly helpful for patients. Previous research supports these observations: HRV-BFB has been shown to reduce arousal in PTSD patients (Lewis et al., 2015), and visual representations of physiological data help patients with anxiety disorders link physical symptoms to psychological causes (Alneyadi et al., 2021).

In terms of implementation challenges, the data reveal heterogeneous but recurring barriers. A major issue is staff shortages due to illness, vacancies, or lack of interest, which are often related to the complexity of BFB systems and the training needed. In addition, there are technical difficulties, such as high acquisition costs and mismatches between purchased equipment and clinical needs. For example, one hospital stopped using BFB after purchasing an inappropriate device. These findings are consistent with those of Schmidt et al., who reported similar barriers to inpatient BFB implementation (Schmidt et al., 2023).

In daily practice, scheduling BFB sessions is often difficult because of the limited staff and overlapping responsibilities. Sick leave and holidays often cannot be covered. Technical problems such as system crashes or inadequate hardware (e.g., limited memory or inaccurate sensors) were also frequently reported. Time constraints emerged as a key challenge. BFB is often not fully integrated into treatment plans and must be fitted between other therapies, making coordination with patients and the team complex and time consuming. As a result, sessions are often postponed or canceled, increasing staff workload and reducing the continuity of care.

These structural issues mirror those described by Deenik et al., who identified time, staff, training, and material shortages as the main barriers to implementing new therapeutic approaches in inpatient settings (Deenik et al., 2019).

Another factor influencing the implementation and delivery of biofeedback (BFB) treatment may be the chosen modality of BFB. The data presented in Table 3 indicate that, with respect to the mean number of patients treated per week, the frequency of sessions per patient stay, and the obstacles that emerge during implementation, the various BFB modalities appear to differ only marginally. However, notable variations in the duration of individual sessions are observed, particularly between EEG-based BFB and other modalities. Hospitals employing EMG-, ECG-, EDA-, or respiration-based BFB reported a wide range of session durations, from less than 20 min to more than 45 min. In contrast, institutions that use EEG-based BFB consistently reported mean session lengths exceeding 45 min.

This increased time requirement within the clinical routine may pose organizational challenges and should therefore be considered when selecting the BFB modality to be implemented. Given that the majority of hospitals surveyed in this study reported using multiple BFB modalities in parallel, the present data do not allow for a differentiated assessment of the specific impact of each modality.

However, at present, there is a dearth of research regarding the influence of different modalities on patient outcomes. As demonstrated in Table 3, the diagnoses for which BFB is applied exhibit only marginal differences across modalities. However, a more thorough examination of this aspect could substantially impact the determination of the most suitable modality for a specific inpatient healthcare context. For example, Stern et al. reported in a pilot study on patients with anxiety disorders that EMG-based BFB improved patients’ ability to relax, whereas the comparison group receiving HRV-based BFB demonstrated increased autonomic regulation and increased parasympathetic tone (Stern et al., 2013). This finding suggests that different BFB modalities may indeed exert distinct effects in the treatment of specific mental health conditions.

Consequently, a comparative analysis of the various BFB modalities may offer significant value, as such an analysis could identify unique characteristics and challenges associated with the implementation of each modality and clarify their specific benefits for particular conditions. Consequently, this approach may allow for a more precise and effective implementation of BFB in inpatient healthcare settings.

Another challenge is related to patient-related factors. Adherence to BFB varied; some patients reported experiencing benefits later in the course of treatment, whereas others were initially skeptical. Some hospitals have emphasized the importance of patient education to address these concerns. Religioni et al. reported that educational interventions can significantly improve adherence (Religioni et al., 2025).

Finally, one of the most frequently cited reasons for not offering BFB was a lack of knowledge among clinical staff. In many cases, BFB was only introduced because individual staff members were already familiar with it. Other barriers included insufficient funding and a lack of adequate physical space. When asked what would be necessary to implement BFB, hospitals most often mentioned increased staffing, appropriate space, and structured training programs.

On the basis of these findings, the following aspects appear to be relevant to ensure successful implementation and transfer into everyday clinical practice. Thorough training and continuous education of therapeutic staff are necessary to guarantee the safe and competent use of BFB technology. Currently, functional BFB equipment and regular maintenance measures are essential to minimize technical malfunctions.

In addition, the implementation and specific application of BFB treatment should be thoroughly planned in advance. Critical points here include the parameters to be used, the professional group to be involved, the duration and frequency of the sessions, and the objective of the treatment.

The required hardware and software as well as personnel and infrastructural resources should be adapted to the requirements to ensure optimal implementation of the therapy. BFB treatment needs to be integrated as an established part of the patient timetable to reduce the degree of organizational effort. Standardized therapy protocols promote consistent implementation and quality assurance. Regular evaluations help patients adapt to therapy. In addition, hospital staff should be comprehensively informed about the scientific basis and potential of BFB to promote acceptance and motivation.

This study provides a broad overview of the use of BFB in inpatient mental health care, specializes in psychosomatic medicine and psychotherapy, and suggests directions for future research. Investigating the relationships among session frequency, duration, parameters used, and treatment areas may help to identify optimal protocols for specific patient groups. The role of complementary exercises (e.g., breathing or relaxation techniques) in supporting the transfer of BFB to daily life should also be explored. Further studies could assess how symptom visualization through BFB improves therapeutic outcomes in inpatient mental health care. In addition, research aimed at optimizing implementation may identify unaddressed barriers and inform strategies to improve the integration of BFB into routine clinical practice.

Several limitations should be considered. First, the participation of the hospitals was voluntary, which introduces the possibility of selection bias. Participating sites may differ from nonparticipating sites in terms of staffing, infrastructure, prior exposure to the therapy, or institutional motivation to implement new treatments. These differences could have led to an overestimation of feasibility or an underestimation of certain barriers. Therefore, the results should not be assumed to be fully generalizable to all German hospitals specializing in psychosomatic medicine and psychotherapy. Additionally, given the voluntary character of the hospital’s participation, it is plausible that the number of respondents in the quantitative section was lower than expected, thereby reducing the representativeness of the findings. As is common in qualitative research (Anderson, 2010), the generalizability of the findings is limited. This limitation is further compounded by the specificity of psychosomatic medicine and psychotherapy to the German healthcare system. This leads to a lack of comparability in inpatient services in other countries without contextual adaptation.

Although psychosomatic medicine is unique in Germany as an independent inpatient specialty, BFB treatment has gained significant international traction, particularly within the domains of psychiatric care and psychotherapy. Therefore, the findings are not limited to the German context, offering transferable insights into the delivery of BFB treatment in routine inpatient care. These insights encompass the challenges encountered, the facilitating factors, and the practical considerations relevant to its implementation in other complex inpatient settings.

Because there is a lack of empirical data on the implementation and routine use of BFB in inpatient settings, the present study provides an initial examination of influencing factors. Future research should prioritize replicating these findings with a larger and more representative sample. Additionally, these studies should evaluate the consistency of the identified barriers and facilitators across different national contexts. Subsequent studies should also examine these factors in other inpatient mental healthcare settings to determine the generalizability and applicability of the findings.

Moreover, the heterogeneity of BFB methods hinders the establishment of a universal framework for comparison. The disparities among the various BFB modalities, or even among the same modalities when employed in different manners, have the potential to exert a substantial influence on the integration and implementation of BFB treatment within an inpatient context. The observed variations in complexity, training requirements, and comprehensibility to patients across modalities are primarily derived from the self-reported experiences of the participating institutions, which may be subject to local practice patterns and individual perceptions. A notable limitation of the present study is its inability to ascertain the rationale behind hospitals’ selection of a specific BFB modality. A comprehensive understanding of these decision-making processes could offer significant insights into institutional priorities, resource allocation, and perceived therapeutic benefits. Therefore, addressing these processes in future research is essential. It is suggested that upcoming studies utilize standardized, comparative designs to examine how different BFB modalities affect implementation feasibility, therapist involvement, and therapeutic outcomes. This approach provides insight into the advantages and challenges of specific modalities and facilitates the targeted and efficient integration of BFB into inpatient mental healthcare.

## Conclusions

BFB is versatile in its areas of application for inpatient mental health care, specializing in psychosomatic medicine and psychotherapy. Although there are many procedural differences between hospitals in terms of the parameters recorded (e.g., EMG, ECG, and ECG), the frequency and duration of the sessions, and the transition to everyday clinical practice, there is also much overlap, especially in reviewing the BFB sessions and treatment progress with patients. In addition, all hospitals reported benefits of the treatment for their patients, possibly through improved self-efficacy, self-awareness, or understanding of their illness. Technical difficulties, scheduling conflicts, and staff shortages are frequently identified as obstacles to the implementation and everyday use of BFB treatment. Specialized training of therapeutic staff, up-to-date equipment, and regular maintenance could ensure safe and efficient use of resources. In addition, permanent integration into patients’ timetables would minimize organizational effort, and sufficient personnel and infrastructural resources would contribute to optimal integration into the clinical routine and high-quality treatment.

## Supplementary Information

Below is the link to the electronic supplementary material.


Supplementary Material 1


## Data Availability

The data supporting the presented results are available upon reasonable request to the corresponding author.

## Abbreviations

BFB: Biofeedback
EMG: Electromyogram
ECG: Electrocardiogram
HRV: Heart rate variability
EDA: Electrodermal activity
EEG: Electroencephalography
NFB: Neurofeedback
PTSD: Posttraumatic stress disorder
RCT: Randomized controlled trial
MMARS: Mixed methods article reporting standards
APA: American psychological association
RF: Respiratory frequency
