# Supplementary material for: Examining the Integration of Biofeedback Treatment in Inpatient Mental Health Care, Specialized in Psychosomatic Medicine and Psychotherapy: A Mixed Methods Study
Source: Appl Psychophysiol Biofeedback. 2025 Nov 21;51(3):511–25. doi: 10.1007/s10484-025-09749-3 (PMC13525012; doi:10.1007/s10484-025-09749-3)
Supplement: Supplementary file 1 [file 10484_2025_9749_MOESM1_ESM.docx]

**Appendix**

**A1** JARS - Mixed Methods Article Reporting Standards (MMARS)

| **Section/**  **topic** | **Item no.** | **Checklist item** | **Reported on page number/**  **line number** | **Reported on section/ paragraph** |
| --- | --- | --- | --- | --- |
| **Title** |  |  |  |  |
|  | 1a | Quant - Identify the populations studied. | Title | Title |
|  | 1b | Mixed - Refrain from using words that are either qualitative (e.g., "explore," "understand") or quantitative (e.g., "determinants," "correlates"), because mixed methods stands in the middle between qualitative and quantitative  research. | Title | Title |
|  | 1c | Mixed - Reference the mixed methods, qualitative methods, and quantitative methods used. | Title | Title |
| **Abstract** |  |  |  |  |
|  | 2a | Mixed - Indicate the mixed methods design, including types of participants or data sources, analytic strategy, main results/findings, and major  implications/significance. | Abstract | Abstract |
| **Introduction** |  |  |  |  |
|  | 3a | Quant - State the importance of the problem, including theoretical or practical  implications. | Page 4 | Introduction |
|  | 3b | Qual - Review, critique, and synthesize the applicable literature to identify key issues/debates/theoretical frameworks in the relevant literature to clarify barriers, knowledge gaps, or practical  needs. | Page 4 | Introduction |
|  | 3c | Mixed - State three types of research objectives/aims/goals: qualitative, quantitative, and mixed methods. Order these goals to reflect the type of mixed  methods design used. | Page 4 | Introduction |
| **Method** |  |  |  |  |
|  | 4a | Mixed -Explain why mixed methods research is appropriate as a methodology given the  paper’s goals. | Page 4 | Methods |
|  | 4b | Mixed - Indicate the qualitative approach to inquiry and the  quantitative design used within the mixed methods design type. | Page 6-7 | Analysis |
|  | 4c | Quant - Report inclusion and exclusion criteria, including any  restrictions based on demographic characteristics. | Page 5 | Study design |
|  | 4d | Qual - Describe the participants/data source selection process and inclusion/exclusion criteria. | Page 5-6 | Participants |
|  | 4e | Quant - Describe settings and locations where data were collected as well as dates of data collection. | Page 5 | Data collection |
|  | 4f | Qual - Provide the general context for the study (when data were collected, sites of data  collection). | Page 5 | Data collection |
|  | 4g | Quant - Define all primary and secondary measures and covariates. | Page 5 | Data collection |
|  | 4h | Quant - Report major demographic characteristics and important topic-specific  characteristics. | Page 6 | Table 1 |
|  | 4i | Quant - Describe procedures for selecting participants. | Page 6 | Figure 1 |
|  | 4j | Quant - Describe agreements and payments made to participants. | Page 5-6 | Participants |
|  | 4k | Qual - State the form of data collected (e.g., interviews, questionnaires, media, observation). | Page 5 | Data collection |
|  | 4l | Qual - Describe questions asked in data collection: content of central questions, form of questions (e.g., open vs. closed). | Page 5 | Data collection |
|  | 4m | Qual - For interview and written studies, indicate the mean and range of the time duration in the  data-collection process. | Page 5 | Data collection |
|  | 4n | Qual - Describe any incentives or compensation, and provide assurance of relevant ethical processes of data collection and consent process as relevant. | Page 5 | Data collection |
|  | 4o | Describe institutional review board agreements, ethical  standards met, and safety monitoring. | Page 7 | Quality Control |
| **Results** |  |  |  |  |
|  | 5a | Quant - Provide information detailing the statistical and data- analytic methods used. | Page 7-10 | Quantitative online  questionnaires |
|  | 5b | Qual - Describe research findings (e.g., themes, categories, narratives) and the meaning and understandings that the researcher has derived from the data analysis. | Page 11-12, page 12 | BFB treatment  process, Themes  discovered |
|  | 5c | Qual - Demonstrate the analytic  process of reaching findings (e.g., quotes, excerpts of data). | Page 13-14, page 14-16, page 16 | Category 1, Category 2, Category 3 |
|  | 5d | Mixed - Indicate how the qualitative and quantitative results were mixed. | Page 17-19 | Discussion |
| **Discussion** |  |  |  |  |
|  | 6a | Describe the central contributions and their significance in advancing disciplinary understandings. | Page 17-19 | Discussion |
|  | 6b | Discuss similarities and differences between reported results and work of others. | Page 17-19 | Discussion |
|  | 6c | Identify the study’s strengths and limitations. | Page 17-19 | Discussion |

Article information:

*As the checklist was provided upon initial submission, the page number/line number reported may be changed due to copyediting and may not be referable in the published version. In this case, the section/paragraph may be used as an alternative reference.

**A2** *Questionnaire quantitative study section*

**1.**

**1.01 Is biofeedback treatment part of your therapy program?**

Yes

No

**2. Yes**

**2.01 In which setting is biofeedback treatment offered at your hospital?**

Inpatient

Outpatient

Both

**2.02 For which disorders is biofeedback treatment used in your hospital?**

Post-traumatic stress disorder

Depressive disorder

Somatoform disorder

Anxiety disorder

Panic disorder

Eating disorder

ADHD

Autism spectrum disorder

Psychosis

Obsessive-compulsive disorders

Further (free space)

**2.03 Are there criteria, that would disqualify a patient from participating in biofeedback treatment at your hospital? How are these exclusion criteria defined?**

Anyone can take part

Lack of compliance

Language barrier

Length of stay too short

Secondary diseases

Limited cognitive abilities

Impaired vision or amaurosis

Hypacusis or deafness

Other (free space)

**2.04 What parameters are collected for biofeedback treatment in your hospital?**

Brain waves (EEG)

Muscle tone (EMG)

Heart rate (ECG)

Skin conductivity (EDA)

Breathing rate

Further (free space)

**2.05 If you use more than one type of biofeedback treatment, which treatment do you use for which condition?**

Free space

**2.06 Which device do you use in your hospital for biofeedback treatment?**

Nexus

Mindfield

Other (free space)

**2.07 Which professional groups carry out biofeedback treatment in your hospital?**

Nurses

Occupational therapists

Physicians

Psychotherapists

Further (free space)

**2.08 Do the therapists have special training for carrying out biofeedback treatment?**

Yes

No

**2.09 Which premises are used for biofeedback treatment?**

A room specially equipped for biofeedback

Patient room

Group room

Occupational therapy room

Single room

Others (free space)

**2.10 How many sessions are scheduled per patient on average?**

1-5

6-10

11-15

16-20

>20

**2.11 How long does a biofeedback session last on average?**

<20min

20-30min

31-45min

>45min

**2.12 On average, how many patients receive one session of biofeedback treatment per week?**

0-5

6-10

11-15

>15

**2.13 Do you follow certain biofeedback protocols when performing it?**

Yes

No

**2.14 If you use different protocols, which protocols do you use? For which disorders you use which one?**

Free space

**2.15 Are there evaluation interviews with the patient before and/or after the biofeedback sessions?**

No

Yes, before each session

Yes, after each session

Yes, before and after each session

Yes, at the beginning and end of treatment

Yes, at irregular intervals

**2.16 What difficulties do you see with the implementation of biofeedback treatment in your hospital?**

Staff shortage

Lack of time

Problems with accounting

Adherence of the patients

Technical difficulties

No suitable premises

Further (free space)

**2.17 Since when has biofeedback treatment been part of your therapy program?**

Free space

**2.18 How is biofeedback treatment accounted for in your company?**

Free space

**2.19 May we contact you for a short interview (by phone or Zoom) to discuss your experiences in more detail and initiate a mutual exchange?**

Yes

No

**3. No**

**3.01 What reasons do you think there might be for not implementing biofeedback treatment in your hospital?**

Lack of knowledge about the use and application areas of biofeedback training

Financial aspects / problems with accounting

Staff shortage

No suitable premises

No corresponding patient clientele

Lack of time

Further (free space)

**3.02 In your opinion, what conditions would have to be met in order to implement a new form of therapy, such as biofeedback treatment, in your hospital?**

Free space

**3.03 Do you think it would be feasible to implement biofeedback treatment in your hospital in the near future?**

Yes

No

**A3** *BFB treatment process summary of the hospitals interviewed*

| **C1** | |
| --- | --- |
| **Used BFB parameter** | Electrocardiography, respiratory frequency |
| **Profession of therapists** | Medical assistant |
| **Introduction to BFB treatment** | The ward will provide an information brochure.  In the first session: Resting heart rate measurement (approx. 5 min), respiratory sinus arrhythmia (RSA) measurement (approx. 1 min deep inhalation and exhalation) and actual BFB exercise. |
| **Performing the treatment** | After connecting to the BFB device, a breathing exercise (abdominal breathing) is performed with the patient, a bar at the edge of the screen indicates the speed, when inhaling it goes up, when exhaling it goes down, each breathing cycle lasts 10 sec, 4 sec inhale, 4 sec exhale and 2 sec pause. A balloon shows how accurately the patient performs the exercise, the balloon rises if the exercise is performed well.  Session duration: up to 10min plus debriefing  Number of sessions per stay: up to 12  Average duration of stay: 6-8 weeks |
| **Evaluation with the patient** | After each session, discuss the bar chart of the current session as well as the progression, with contextualization. |
| **Transfer BFB to patient’s day-to-day life** | The patient is encouraged to practice the breathing exercise outside the BFB (3 times/day for 3 minutes), in an environment that is comfortable for the patient, if possible. Later also in tense situations, i.e. regulation skill. |
| **C2** | |
| **Used BFB parameter** | heart rate variability |
| **Profession of therapists** | Nurse |
| **Introduction to BFB treatment** | Introduction to the device for independent practicing. |
| **Performing the treatment** | Measurement of heart rate variability using a finger sensor, BFB device is held in the hand, the device specifies a breathing rate according to which the patient should breathe. The device changes color from red to green when the exercise is performed correctly. Exercise duration 7min.  Session duration: 7min  Number of sessions per stay: variable (Independent training, patient decides when and how often to use the tool)  Average duration of stay: at least 6 weeks |
| **Evaluation with the patient** | Inquiry by the reference nurse about frequency of use and subjective effect of the exercise on the patient (rather irregular). |
| **Transfer of BFB to patients’ day-to-day lives** | No information or no transfer. |
| **C3** | |
| **Used BFB parameter** | Electromyography, respiratory frequency |
| **Profession of therapists** | Nurse |
| **Introduction to BFB treatment** | Preliminary discussion about previous experience, expectations and brief medical history.  Introductory discussion, recording of muscle tension via EMG at various points (especially neck area) to determine the current status and to introduce patients to the BFB, followed by stress testing (patient is deliberately put in a stressful situation, e.g. through mental arithmetic tasks or knowledge tests, to record changes in muscle tension in stressful situations). |
| **Performing the treatment** | Muscle (tension) relaxation training, discrimination training, breathing training, depending on the clinical picture (exact procedure not explained in detail). The patient sees the derived derived graphical feedback as feedback and receives an explanation of their meaning during the session, and individual instructions on how to move the derived parameter into the desired range.  Session duration: 25-50min  Number of sessions per stay: 6-8  Average duration of stay: 8 weeks |
| **Evaluation with the patient** | Discussion of the initial session at the beginning of the first regular session. Interactive discussion of the individual sessions, elaborating on the assessment of the patient and the therapist, as well as the patient's needs, uncertainties and successes. Final discussion in the last session, to evaluate the entire course of BFB treatment and the patient's development throughout it. |
| **Transfer of BFB to patients’ day-to-day lives** | “Micro homework” 2x/day three rounds, tensing and releasing, while tensing imagine that you are tensing half a centimeter.  In the later sessions, BFB is left out from time to time, so that the patients have to concentrate completely on their own perception. |
| **C4** | |
| **Used BFB parameter** | Electroencephalography |
| **Profession of therapists** | Medical assistant |
| **Introduction to BFB treatment** | Introductory discussion about the learning theory content, effects and procedure of the BFB. |
| **Performing the treatment** | Measurement of brain waves (slimmed-down EEG), the aim is to keep the patient in a brain wave range that is associated with relaxation. The feedback is an image that becomes clearer when approaching the desired brain wave area and blurrier when deviating from the area.  Session duration: 45min  Number of sessions per stay: 6-12  Average duration of stay: 8 weeks |
| **Evaluation with the patient** | In conversation with the patient (not explained in detail). |
| **Transfer of BFB to patients’ day-to-day lives** | No information or no transfer. |
| **C5** | |
| **Used BFB parameter** | Electrodermal activity, Electromyography, Respiratory frequency |
| **Profession of therapists** | Ergotherapist, Physiotherapist |
| **Introduction to BFB treatment** | Introductory talk about the method, skin conductance insertion measurement. |
| **Performing the treatment** | Short conversation at the beginning about where the patient is currently experiencing discomfort or tension in the body, whether there are stressful issues that are currently at the forefront. Various types of relaxation training (not explained in detail) in which a picture becomes clearer or pleasant music is played when the patient is in the desired measurement range. Breathing training in which the BFB device guides the patient by means of a graph, duration of inhalation, duration of exhalation, brief pause, new cycle.  Session duration: 50min  Number of sessions per stay: variable, depending on the patient's needs (on average, once a week)  Average duration of stay: no information |
| **Evaluation with the patient** | The last 15 minutes of each session, review of the data and graphs of the current session, with explanation and assessment by the therapist and reference to the topic / complaints from the conversation at the beginning of the session. |
| **Transfer of BFB to patients’ day-to-day lives** | No information or no transfer. |
| **C6** | |
| **Used BFB parameter** | Electrodermal activity |
| **Profession of therapists** | Psychotherapist |
| **Introduction to BFB treatment** | An information brochure is provided by the station.  Detailed explanation of the procedure, device, parameters, etc. and stress testing (similar procedure as described in C3) as an introductory session. |
| **Performing the treatment** | Described as behavioral therapy with visual support.  Three phases: Short conversation about the patient's current situation, issues that are in the foreground, current complaints. After connecting to the device, 5-minute baseline recording, during which the patient sits quietly for 5 minutes and breathes in a relaxed manner. Then the action phase, during which the patient should actively deal (mentally) with situations that are stressful for him and thus trigger a stress reaction. This involves looking at the screen and reflecting on which thoughts lead to an exciting and which have a calming effect. This is followed by a 10-15 minute relaxation phase. When changing phases, the subjective level of tension is assessed (on a numerical scale from 1-100).  Session duration: 50min    Number of sessions per stay: up to 8  Average duration of stay: 10 weeks |
| **Evaluation with the patient** | Short evaluation at the phase change, a look at the graph at the end of each session and reflection on the session, particularly emphasizing progress in relaxation, emotional naming and formulating helpful thoughts. |
| **Transfer of BFB to patients’ day-to-day lives** | Patients are given a log in which they are asked to describe stressful situations that will be discussed at the next appointment in the action phase. When taking notes, patients should particularly practice naming situations and the emotions and thoughts that arise. Patients should also keep a relaxation diary in which they write down what helped them acutely when they were tense, discussed at the beginning of the next session. Patients are also given exercises to direct their attention, that they can do independently. |
| **C7** | |
| **Used BFB parameter** | Heart rate variability, others depending on the disorder (not explicitly mentioned) |
| **Profession of therapists** | Psychotherapist, medical assistant |
| **Introduction to BFB treatment** | An information brochure is provided by the station.  An explanation of the procedure and the parameters that are collected during the BFB. Stress diagnostics, in which the patient is exposed to various potentially stressful situations, such as mental arithmetic, reciting animals with Z, reciting a poem or singing a song. Several parameters are recorded, including muscle tension via EMG, body temperature, pulse, etc. |
| **Performing the treatment** | During the session, the patient sees the derived graphical feedback, which they can use for orientation. During the session, the therapist repeatedly focuses on the patient's self-perception, e.g. by asking specific questions about where the patient is currently experiencing tension or what thoughts are currently arising.  Session duration: 50-60min  Number of sessions per stay: up to 6  Average duration of stay: 13-17 weeks |
| **Evaluation with the patient** | Follow-up discussion after each session, with specific questions about the patient's self-perception during the session. |
| **Transfer of BFB to patients’ day-to-day lives** | In the course of the therapy, the screen is covered for the patient to practice body awareness, even without the feedback. Various exercises to be carried out outside the sessions, depending on the patient's symptoms, e.g. breathing exercises. |

**A4** *Quotes of themes and subthemes*

| **Obstacles to implementing BFB treatment** | |
| --- | --- |
| **Structural obstacles** | |
| **Lack of employees** | [Q1] „You then also need the personnel who want to carry it out.“ (C4) |
|  | [Q2] „There was also the idea of doing this with the nursing staff. However, due to their sick leave, rotation, staff shortages and deductions, it was unfortunately not possible for us to facilitate more training sessions without the psychologist.“ (C6) |
|  | [Q3] „And because we want nursing to take over some of the BFB sessions as well,[....] with the situation that we didn't have all the nursing positions filled, they were part-time positions and nobody really wanted to feel responsible for them from nursing.“ (C2) |
| **Technical equipment barriers** | [Q4] „At first, it wasn't so easy to identify the right electrodes. We looked around for the right software and who was using what? So practically finding the right module and the right leads.“ (C3) |
|  | [Q5] „Hurdles. The device with which we wanted to use the EMG in pain patients is a totally complicated device, which can and should also be used for scientific questions.“ (C2) |
|  | [Q6] „Of course, buying such a device is always a matter of course. I think the Neurom costs four and a half thousand. If you buy the simplest version of the Nexus, you're also looking at €3,000-4,000. Of course, that's an investment you have to make.“ (C4) |
| **Process obstacles** | |
| **Training of therapists** | [Q7] „So the biggest hurdle was certainly training an employee.“ (C4) |
|  | [Q8] „And that's why it needed such a complex introduction. And because we wanted the nursing team to take over part of the biofeedback session together with the patients. We had the feeling that this introduction was somehow not feasible. Precisely because it is too complex.“ (C2) |
| **Long implementation phase** | [Q9] „There are so many slow processes, I would like to see them a bit faster.“ (C4) |
|  | [Q10] „Yes, one and a half to two years. So I came here two years ago and then, I think, after three months or so, it was somehow clear that we wanted to revive it and then, until we had the momentum, who was going to do it, had decided who was going to do it and who was going to do the training. And by the time we had the device up and running [....] So yes, the preliminary work and a room set up.“ (C5) |
| **Everyday clinical practice transition** | [Q11] „It was a bit of a hurdle that the colleagues who were trained were psychological psychotherapists working here, and they somehow didn't manage to fit it into their everyday work.“ (C5) |
|  | [Q12] „And the original concept was that each of them should receive biofeedback. We're not implementing that at the moment. But that's what we really wanted to do. And then we also hit the organizational difficulties.“ (C6) |
| **Obstacles for everyday clinical practice** | |
| **Structural obstacles** | |
| **Staff shortage** | [Q13] „Then there are personnel-related reasons, that we are not always fully available and then the psychologists and psychotherapists who are also involved in biofeedback simply have to substitute other things that take priority.“ (C6) |
|  | [Q14] „The employee then also has other commitments. She doesn't have as many slots.“ (C4) |
|  | [Q15] „Only I actually do that. If I'm ill or on vacation, then it doesn't happen.“ (C1) |
|  | [Q16] „What makes it rather difficult is that when I'm on vacation, my biofeedback is down.“ (C3) |
|  | [Q17] „And since then, unfortunately, we have been struggling with massive staff shortages, just like last week, so that we haven't really recovered in terms of biofeedback since corona.“ (C7) |
| **Technical hard- and software barriers** | [Q18] „And my colleagues also say that it somehow doesn't work reliably. And then, um, well, I have the impression that there are hurdles among colleagues, especially when it comes to technical implementation.“  (C2) |
|  | [Q19] „In the beginning, the whole thing was like an old tablet [....] you could only take five or eight patients at a time. You had to keep deleting them. So until. So when they were finished, you could put someone new on it.“ (C1) |
|  | [Q20] „Technical difficulties sometimes. We had an old device where the battery had to be charged and then it didn't stand properly on the station. Or the technology itself was no longer correct with the sensor.“ (C6) |
| **Access to premises** | [Q21] „It often has a bit to do with charm. Doing it when you don't have an area somewhere where you can do it without being seen.“ (C2) |
|  | [Q22] „That the room in which the device is located may not be occupied by other therapies.“ (C6) |
|  | [Q23] „[….]so that at least we are no longer tied to the room, but always had to walk around with our laptop, bag and suitcase.“ (C6) |
| **Process obstacles** | |
| **Training of new therapists** | [Q24] „Otherwise, when there is a change of personnel, the new colleagues have to be trained again and again.“ (C6) |
|  | [Q25] „And we are possibly already planning to take one or two doctors or psychologists with us. But it's usually difficult to leave it in their hands because they change frequently.“ (C5) |
| **Lack of integration into patients’ timetables** | [Q26] „Each group has a different therapy plan and then I have to see when each group has a gap. I can pick them up in that time, but if there's still um individual therapy, which is something that always wants to be included in these gaps, then they still go first.“ (C1) |
|  | [Q27] „So if the patients now have an individual consultation [....], the other therapies, they always take precedence somehow.“ (C1) |
|  | [Q28] „Hm, yes, I'll say it should be about twelve units. And the biggest difficulty is certainly getting the schedule right.“ (C4) |
|  | [Q29] „The biggest difficulty is usually to file it organizationally.“ (C6) |
|  | [Q30] „It really has to be said that this has not yet been implemented very well in the treatment plan itself. Unfortunately, it has to be said that it's actually more of a sideline.“ (C4) |
|  | [Q31] „But we keep forgetting because we don't have a fixed protocol for it. That's our problem.“ (C2) |
| **Patients-therapy-relationship** | [Q32] „I had two patients who had past experiences with the Stasi. They were very, very skeptical until the third appointment, they really needed time to gain trust and then to be able to engage with their own content.“ (C6) |
|  | [Q33] „[…] that anxious patients in particular, when they are suddenly turned into cyborgs with all the electrodes and sensors, react with a certain amount of anxiety.“ (C7) |
|  | [Q34] „Because many people find it strange when they suddenly have lots of cables around them and see graphs and values.“ (C5) |
|  | [Q35] „But you also have to pay attention to some of them. They're already distracted again, looking at what the balloon is doing up and down. So no, then you sometimes have to tape it off so that they really only concentrate on the bar.“ (C1) |
|  | [Q36] „Perhaps this is not done so consistently, because a yes could have something to do with resistance to therapy in general. Would be possible.“ (C2) |
| **Considerations** | |
| **Medication Effects** | [Q37] „I'll take a look at how you select the medication list. After that, of course, you have to interpret the parameters differently.“ (C3) |
|  | [Q38] „So with heart rate variability in particular, we naturally have all the beta blockers, which simply make the system a bit rigid.“ (C7) |
|  | [Q39] „Of course, as the doctor in charge, I also have the medication in my head a little bit, so the effects of medication on biofeedback are of course important. So, if the increase is perhaps not so great, because the beta blockers are dosed improperly or something similar. That always makes sense, doesn't it? I've noticed that several times. If we then changed something in the medication, something definitely changed in the biofeedback.“ (C7) |
| **Effects of BFB treatment on patients** | |
| **Increase in self-efficacy** | [Q40] „There are some people who say “Wow, that's really great, I relax there. I haven't experienced that for a long time.““ (C4) |
|  | [Q41] „And that this patient was suddenly experienced as being a lot more relaxed in everyday life, including in the ward rounds, where it was also mentioned that the nursing staff, for example, reported back that the patient came to the nursing staff less to reassure herself or to express her inclinations.“ (C5) |
|  | [Q42] „Many patients then also say, „yes, that has helped them to feel what it's like to relax“, because in our relaxation group [....] Well, they don't have direct feedback at the beginning. They don't have the curves, they have to rely on their body feeling right from the start. And that can sometimes be a bit difficult.“ (C7) |
|  | [Q43] „For example, someone was in the forest and had a panic attack. And then he said he sat down on a tree stump and then first thought about breathing and then always managed to get himself out of the situation.“  (C1) |
|  | [Q44] „At the anniversary, I saw the woman again who is still doing the exercises. “I did ten years ago and that helps me, it's amazing.“” (C3) |
| **Improvement of body-awareness** | [Q45] „And then you notice from week to week how the patients simply get a better feeling and no longer tense up so much.“ (C3) |
|  | [Q46] „Muscle tension thing, where you can, so to speak, help people who have neck pain or who are constantly tense, so that they register what it's doing to them in the first place, and that's often a valuable insight.“ (C5) |
|  | [Q47] „In the case of anxiety patients, they also realize that their body is going up, but there is also a calming ability that they can take with them as feedback.“ (C6) |
| **Improvement in illnes-introception** | [Q48] „For example, talking about muscular tension in chronic pain patients, even though the patients initially don't perceive this at all in their self-perception, but then have very high values in the muscular discharge. Yes, and then it is possible to communicate this, which is then more acceptable for the patients.“ (C3) |
|  | [Q49] „It would be more difficult to talk about it without this tool. „Look, we have very high tension levels here. That's funny. You don't experience it personally. On the other hand, you have whole-body pain. Can we talk about whether there are any connections?““ (C3) |
|  | [Q50] „She always says it feels so strange and she has always found it difficult to describe. And with her, we have actually been able to achieve through biofeedback that she has recognized this for herself. “I'm not as tense as I always thought”. But a lot of things, such as a tendency to brood, have played a role in the negative sense.“ (C5) |
|  | [Q51] „So what we also had as therapy from two patients was that they were very impressed by how blatantly psychological processes can also be depicted physically. Yes, that you can see it as a number, and in the curve, yes.“ (C5) |
|  | [Q52] „Once in the development of the individual disorder model, especially in the area of somatoform disorder. That is very true. There can be connections between the psyche and the body, where every now and then there is a nice aha effect.“ (C7) |
|  | [Q53] „I just love to see how amazed the patients look. „Wow, if I lift my shoulder now, something will happen. And when I think back to last weekend, which was just a huge disaster, which was just so bad for me, bang, something happens on the screen too. How can that be? Couldn't there be a connection between what I think, between what I feel and perhaps physical expressions?“ So that's an aha effect.“ (C7) |
| **Delayed impact of BFB** | [Q54] „But like every form of therapy, not every therapy is suitable for every patient. Some benefit more from one and less from the other. I think that's only fair if you can also attribute this to biofeedback.“ (C7) |
|  | [Q55] „However, for many, the effect only sets in a few weeks after the end of training. At least that's our experience.“ (C4) |
|  | [Q56] „With other patients, they are still very. Even with four to five appointments [....], they are still very dependent on me, especially when it comes to naming affect.“ (C6) |
